# On the Frontline—A bibliometric Study on Sustainability, Development, Coronaviruses, and COVID-19

**DOI:** 10.1007/s11356-021-18396-0

**Published:** 2022-03-06

**Authors:** Andrea Gatto, Carlo Drago, Matteo Ruggeri

**Affiliations:** 1 Wenzhou-Kean University, CBPM, Wenzhou, 325060 Zhejiang Province China; 2 Natural Resources Institute, University of Greenwich, Central Avenue, Chatham Maritime, ME4 4TB UK; 3 Centre for Studies on Europe, Azerbaijan State University of Economics (UNEC), Baku, Azerbaijan; 4 University of Rome N. Cusano, Via Don Carlo Gnocchi 3, 00166 Rome, Italy; 5 Istituto Superiore di Sanità, Viale Regina Elena, 29900161 Roma, RM Italy; 6 St. Camillus International University of Health Sciences, Via di Sant Alessandro, 8, 00131 Roma, RM Italy

**Keywords:** COVID-19, Sustainability, Development, Coronavirus, Pandemics, Bibliometrics, C0, I15, I18, Q01, Q56

## Abstract

The COVID-19 pandemic has placed the world’s population in a state of unprecedented public health and global health vulnerability. Risks to public and global health have escalated due to COVID-19 contamination. This has raised the statistics of inequity and environmental concerns. A possible outlook entails reducing the pandemic consequences by prioritizing development, biodiversity, and adaptability, offering buffer solutions. It contains vital methods for studying, comprehending, and unraveling events—examining early responses to COVID-19, sustainability, and development, relating them with overall Coronaviruses reaction. This study maps out environmental, socioeconomic, and medical/technological issues using as statistical techniques multiple correspondence analysis and validated cluster analysis. The findings encourage rapid, long-term development policy involvement to address the pandemic. The resulting crises have highlighted the necessity for the revival of health justice policies anchored in distinctive public health ethical patterns in response to them. As a general rule, resilience and preparedness will be targeted at developing and vulnerable nations and are prone to include access to vaccines, public health care, and health investment. Our findings show the relevance of innovating on sustainable development routes and yardsticks. Sustainable global health requires crucial measures in prevention, preparation, and response. Long-term policy recommendations are needed to address pandemics and their interrelated crises and foster sustained growth and socioecological protection.

## Introduction: Coronavirus and pandemics disentangled—The increasing need for sustainability and development trajectories

Pandemics and infectious diseases hinder worldwide development (Jonas, 2013; Brahmbhatt and Dutta, 2008; WHO, 1996; Becker, 1990). Pandemics have been causing grand socioeconomic and ecological disruptions, hampering inequalities and poverty, especially among people living in extreme poverty (Loayza and Pennings, 2020; Sumner et al., 2020; Hofrichter, 2003; Benatar, 2002). This evidence confirms that unequal societies are more likely to hamper population health issues, wellbeing standards and further socioeconomic factors (Pickett and Wilkinson, 2015). Major epidemics and pandemics have hit specific regions or worldwide vulnerable people from both the Global North and South throughout the last century (Ho and Gatto, 2021). Some of the harshest outbreaks include infectious diseases and Coronaviruses such as the Spanish flu, the 1957-1958 Asian influenza, the Ebola virus group, the SARS, and COVID-19. Conversely, from the former, the 2019 Coronavirus is different for its community spread and severity—factors changing daily life and affecting both short- and long-term development (WHO, 2020a).

Public, global, and environmental health is intertwined with sustainability and development (Di Marco et al., 2020). This is particularly true for resource governance, vulnerability, and resilience, where protection from food, energy, and water shocks turns nodal (Gatto and Drago, 2020a; Morrow et al., 2019; Melo and Gatto, 2014). Vulnerability to shocks and major events are likely to affect complex systems (Gatto and Busato, 2020). Similarly, vulnerability to pandemics must be understood through an overarching view on global complex systems, contemplating institutional instability (Benatar, 2002). Monitoring the repercussions on the vulnerable is paramount to understanding the likelihood of falling into socioeconomic traps.

This aspect is particularly frail for the global consequences that might be reflected on the global macroeconomic front (Abel and Gietel-Basten, 2020). The outbreak notably put the private sector into a deep crisis—although some sectors have benefited from the pandemic. According to the business sector and the specific company response, industry has been hit and reacted in different ways in terms of strategies and production changes (Bapuji et al., 2020).

Geographic proximity and location have shown themselves to deserve thorough and analytical academic investigation. This involves the study and visualization of contagions spread (Zhou et al., 2020), countries and modalities of the actions to tackle the virus (Wei et al., 2020), as well as the social distancing and people's movements dynamics (Zhai et al., 2020), and migrant workflows (Abel and Gietel-Basten, 2020). All these spatial aspects return exceptional socioeconomic inequality results relevant for COVID-19 development research and policy investigation.

Food and nutrition security encase overriding and delicate sectors during pandemics and infectious disease outbreaks, and COVID-19 is no exception (Phillipson et al., 2020). These affect both supply and demand sides and hit diverse geographical locations, as well as the health and socioeconomic possibilities of the foreign and migrant agriculture workers (WEF, 2020). Food must be framed into complexity, where useful, valuable data and information are required to deliver solid policy and decision making (Mock et al., 2013).

Food sustainability is a determinant factor in ensuring development and environmental and global health, facilitating major significant consequences, alleviation and smoothing risk, and adverse events (Civero et al., 2021; Agovino et al., 2018). Above all, food production and value chain practices have been found to be focal amidst the COVID-19 occurrence (Cattivelli and Rusciano, 2020). They are likely to come up with the opportunity of delivering fungible applications for a food transition envisaging sustainable consumption patterns (Cohen, 2020).

The scientific proliferation in the novel Coronavirus disease confirms the importance of detecting the research dynamics amidst the outbreak (Duan et al., 2020). It is of primary importance disentangling cooperation networks, scholars, and publication results concerning the geographical location.

This research aims at analyzing existing publications on the emerging COVID-19, connecting them with sustainability and development research topics. The work in hand exploits an up-to-date bibliometric analysis on the publication records on these themes to investigate this nexus. We could exploit quantitatively the extensive literature in the field by considering exploratory statistical techniques. In this vein, we provide visual quantity representation of scientific research on the virus, as well as its influence on the environment to offer the decision-makers relevant insights that can be used in policymaking. We attempt to link COVID-19, Coronaviruses, sustainability, and development starting from the existing literature. The final goal is to raise awareness, enhance comprehension of the available facts, and contribute to sustainable development research to expand knowledge and concentrate viable solutions. The work also drafts policy recommendations for the way forward, rooted in resilience action, provides preparedness for upcoming pandemics and global health disruptions

The article is organized as follow: in section 2, we describe the previous relevant literature on the resilience preparedness and mitigation for the vulnerable; in section 3, we present the bibliometric dataset and the methodologies used; in section 4, there is the description of the statistical results wherein the fifth section there is a discussion on the social and the policy implications of the work; finally, in section 6, the conclusions are drafted. Three appendixes also accompany the article: on the complete tables (A), on the clustering of the different keywords and topics related to the literature (B), and, finally, on multiple correspondence analysis (C).

## Navigating the unknown—Resilience preparedness and mitigation for the vulnerable

As for precedent viruses, the COVID-19 disease is likely to have originated from zoonotic spillover—infections and transmissions coming from animal species (Zhang, 2020; Jones et al., 2008). This fact raises significant concerns about biological hazards and pandemics and calls for resilience preparedness and systemic view for international governance (Djalante et al., 2020; FAO 2008). As a massive-scale ongoing pandemics episode, the 2019 Coronavirus is a prime adverse event necessitating holistic perspectives and systemic outlooks to foster resilient and sustainable governance intervention (Gatto, 2020). Tackling this unexpected situation is a challenge that our society is urgently required to govern for preserving sustainability (Pirouz et al., 2020). Novel policy responses to pandemics demand sustainability action to adapt and socioeconomic, ecological, and institutional downturns. The process is mutual: on the same wavelength, the sustainable development agenda needs to adjust its priorities and consider the possibility of upcoming pandemics, easing global health programs (Di Marco et al., 2020; Bogich et al., 2012).

Poor and vulnerable people are the most exposed to (natural) hazards (World Bank, 2012). They are the most likely to be hit by infections and significant disease and experience severe socioeconomic consequences, structurally worsening their life and health conditions, and die. The most affected categories include women, the elderly, people with disabilities and illnesses, and minorities at risk, principally located in remote rural areas of the Global South (Bapuji, 2020; Lloyd et al., 2020; Loyaza and Pennings, 2020; Sumner et al., 2020). The COVID-19 contagions and consequences risk becoming a plague in developing countries and for the migrants due to the frequent inadequate levels of public health and social nets, lack of preparedness, multifaceted vulnerability, and further systemic deficiencies that they are often connotated by (Gilbert et al., 2020; Loayza and Pennings, 2020; Abel and Gietel-Basten, 2020).

When it comes to unfolding pandemics impacts, the economy-health nexus turns out to be paramount. This aspect also reflects the significance of health within the broader development. It is no news that health dynamics—summarized as life expectancy—is one of the three pillars of human development, along with the economy—GNI—and education—schooling (UNDP, 2010). The importance of entailing the capability approach to investigate pandemics and find sustainability and development policy recommendations therefore arises. Human development established holistic outlooks to explore development and is strictly linked to sustainable development due to its multifaceted perspective and inter-generational equity component (Cisco and Gatto, 2021; Gatto, 2020). Human development propensity to strive for reducing vulnerability through resilience policies and practical action (Malik, 2014) underlies the interconnectedness between development, sustainability, and pandemics. It paves the way for global access to health and health justice (Venkatapuram, 2013).

## Methodology: Bibliometrics and Conceptual Structure Map of the relevant themes

The scope of bibliometrics is to examine the relevance and the impact of published works (Iftikhar et al. 2019). A bibliometric analysis helps evaluate the research trends, the most relevant topics on a research corpus, and the cooperation networks between different authors. P﻿revious studies have assessed bibliometric explorations' effectiveness to summarize large research corpora, define, examine, and disseminate complex phenomena (Drago et al., 2021; Gatto and Drago, 2020b; Ho and Gatto, 2021). Bibliometrics is particularly worth portraying scientific advances and delineating the main undertaken guidelines (Aria and Cuccurullo, 2017). The method is also useful for identifying and discovering new research insights from actual trends, being worth synthesizing the existent research and mapping the actual knowledge on a specific topic (Zhang et al., 2019; Cobo et al., 2015).

Bibliometrics is becoming increasingly popular and is being used to evaluate research outcomes more frequently (Wallin 2005). In this study, we hope to gain a better understanding of the fields of COVID-19, the Coronavirus family, Sustainability, and Development. At the same time, we also assess its current level of advancement as literature. We provide a comprehensive picture of the literature's development (Shi and Li, 2019). More general use of bibliometric approaches is extracting and identifying the most relevant subjects or general literature from citations in a literature corpus. When selecting relevant literature in the Big Data era, this technique is essential—bearing in mind the exponential growth of the literature on COVID-19, which needs adequate analysis (see Drago and Hoxhalli, 2020; Dieguez Campa et al., 2020). Furthermore, examining the significant relationships between the various issues at the same time may yield useful insights for future research. Additionally, bibliometric analysis can be used to critically evaluate and compare the results of different studies, allowing for the combination of different findings. In this regard, bibliometric analysis is critical in assisting researchers in their study efforts (Ho and Gatto, 2021).

The logic of bibliometric analysis is, hence, considering existing findings from the actual research and map the possible inter-relationships between the topics. Thus, it is possible to discover new insights and discoveries from these clusters or groups on the existing literature.

More specifically, this study examines COVID-19 and Coronavirus research related to sustainable development and sustainability challenges. In order to conduct a full bibliometric study to examine the COVID-19 themes and sustainability concerns, the bibliometric database was divided into three independent queries on the SCOPUS database. Each query contained a single COVID-19 topic and sustainability issue. The three queries that were utilized are as follows:

1. "COVID-19" AND "Sustainability" AND "Development";
2. "Coronavirus" AND "Sustainability" AND "Development";
3. "COVID" AND "Sustainability" AND "Development".

The most recently acquired queries were resorted from the SCOPUS database. The essential strings were searched for from 2003 to 2020. In particular, we wanted to get a global picture of COVID-19 and Coronavirus and sustainability, and we could not find any previous research that elaborated a similar exercise. The inspected works from the selected queries can be considered the data sample used in this research. From the strings, we have collected the scientific works from the SCOPUS database, and each data (which can be defined as “citation data”) is related to the whole bibliographical information of each work.

The statistical methodology can be described as follows: from the initial bibliographical dataset constructed using the different queries, we perform a descriptive and an exploratory data analysis of the different data sources. Secondarily, we elicit the multiple correspondence analysis evaluating the keywords indicated by each study's authors. We obtain a "conceptual structure map" from the multiple correspondence analysis to project the various keywords. Finally, to identify the relevant groups of keywords in the literature and their relationships, we can cluster the different groups of keywords. From the analysis of the diverse clusters, one can better interpret the existing relevant trends on the literature and the relationships between the keywords and literature themes . This means to examine the keywords which stand at a lower distance means the existence of a work containing both.

We base our decision on the research issue: *In what ways may a global crisis affect sustainability and development when researching this field?* First and foremost, there is a relevant literature gap on this topic (Leal Filho et al. 2020). Following that, we select the distinct keywords based on the research question: *When exploring this sector, how does the global financial crisis affect sustainability and development?* Our goal is to examine the studies on sustainability and development from a scientific perspective. According to Keshky et al. (2020), COVID-19 is likely to harm the quality of life, global political and environmental sustainability, and global economy in a long-lasting time frame; all of these factors will hurt the future of the planet and the human population.

In this paper, we want to examine the influence of Coronaviruses and COVID-19, also known as Coronavirus disease 2019, on sustainability and development. Choosing the considered time span is significant because, by analyzing the literature from this period, we will be able to use our findings to examine the late development of the literature from a distinct phase of the crisis's evolution. This is an innovation with respect to most of the field publications, which only consider COVID-19 instead of including Coronaviruses as well.

The paper is primarily concerned with COVID-19 because the analyzed publications on the other items returned in the string performed has remained largely not relevant. Meanwhile, we are investigating the Coronavirus family and COVID-19 in terms of their long-term viability and development. In order to better comprehend the evolution and impact of COVID-19, it is vital to accurately understand the transmission, survival, and ability to evolve the SARS-CoV-2, according to Zhan et al. (2020) and Matheson and Lehner (2020). The environmental science viewpoint broadens our ability to understand the phenomenon and provides us with the opportunity to explore this approach to sustainability and development in order to better understand the causes of and to imagine suitable reactions to the COVID-19 pandemics.

The collected data covered all the variables investigated—authors of the works, titles, year of the study, publishing journal, and further publications specifications. The three bibliometric data collections were combined in a unique database as prescribed by chief scholarship (Moed, 2012). The final corpus obtained by merging the different works related to the queries represents the aim to study the relationships assessed. A primary reason for combining various bibliometric databases is the possibility of getting several queries—that for this study turns to be complementary. In this way, an attempt is made to consider the bibliometric information available without excluding the main lines of research and avoiding constraints.

In order to perform the statistical analysis of the bibliometric dataset collected, a two-step analysis has been undertaken. In the first part, the data was analyzed using an exploratory data analysis. In the second phase, the structure of the sectorial bibliometric research was investigated to reach the relevant "core" of the literature (see Aria and Cuccurullo, 2017). A statistical analysis of the principal findings was performed in the first moment—including the literature as the citation analysis, the most prolific authors, the most associated and recurrent keywords and topics. Then, the dataset was transformed by examining the works characterized by the different keywords. In order to analyze these data, the co-citation network was assessed. The latter was analyzed by using a multidimensional data analysis. This technique allowed to extract the pertinent clusters to identify the general statistical cores of salient literature. More importantly, this step is crucial to understand the cardinal relationships between the highlighted concepts within the literature.

Diverse health policies can be assessed, to be extracted from the health costs and benefits literature. It is also compulsory to assess other types of keywords and analyze the various alternatives in the literature. A leading fact to be pointed out is that the bibliometric analysis does not necessarily substitute the systematic literature reviews in constructing the cost-benefit models in health economics. Bibliometric analyses unveil the core themes and linked works. Hence, it is possible to complement the systematic review for building the health economics models (see Drummond et al., 2015 for the classical construction of health care programs evaluation models). At the same time, the bibliometric analysis can improve the elaboration of health economics models by allowing exploring different specifications. The complete results, related to the techniques and methodological steps undertaken, are visualized in detail in Appendix A (Table 1).

In Appendix C is described the statistical technique performed in the bibliometric analysis: the multiple correspondence analysis (Greenacre and Blasius, 2006; Gherghi and Lauro, 2004; Gilula et al., 2001; Husson, 2021; Guttman, 1941).

**Fig. 1﻿:**
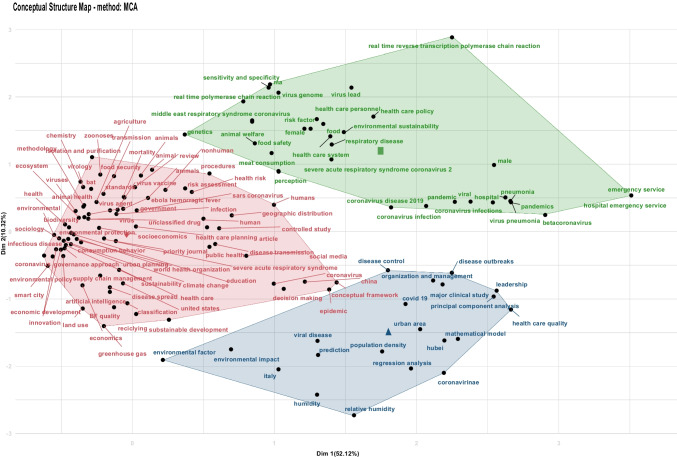
MCA procedure with clustering validation (3 clusters identified). In Figure 1, the x-axis is related to the first dimension extracted from the Multiple Correspondence analysis, whereas the y-axis is related to the second one. The total inertia (variation) included within the data is in parenthesis. Appendix D  (Table 2) shows the content and the interpretation of each cluster.

**Fig. 2:**
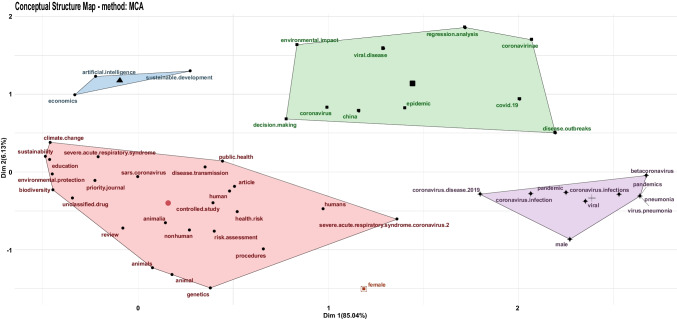
Robustness check: 5 clusters.

Previous scholars have mapped research networks on the 2019 Coronavirus (Duan et al., 2020). The study in hand used a second technique to depict current publications on COVID-19, Coronavirus, development, and sustainability. Namely, multiple correspondence analysis and validation clustering exercises on key terms were performed. This step was necessary to provide a clearer picture of trend research topics on the issues—represented within the first cluster. The second cluster has been used to corroborate the precedent results and analyses. The figures and details of the conceptual cluster maps can be found in Fig. 1, Fig. 2, and Appendix B.

## Results: Key points—What is scholarship showing?

From the primary information about data, it is possible to identify some descriptive information on this strand of research. It is fundamental to sketch the number of documents (300), statistical sources (168), keywords (817) used in the literature covering a period of 17 years (2003-2020).

The average citation for the documents is 9.301. The second part of these statistics allows to better understand the characteristics of the cooperation in the analyzed literature: there, 35 authors of single-authored documents; 806 authors of multi-authored documents; and 52 single-authored documents emerge. From these statistics, it is also possible to compute some relevant indexes: the number of documents per author is 0.364, whereas there are 2.75 authors per document and a cooperation index of 3.17.

This study took into account existing publications on different Coronaviruses, highlighting sustainability and development publications. Due to the more significant impact of COVID-19, the larger number of overall worldwide scholarships, and increasing research interest in various interconnections of the former with sustainability and development studies, the scientific production of these works strongly increased from 2019 to the first part of 2020.

In particular, in 2020, there was an increase to 10 times that of scientific production with respect to 2019. The publication products are mostly research articles (186), but 56 reviews shall also be noticed. The most prolific authors are Haghshenas SS (12 articles); and Gautam S and Pirouz B (9 articles per each). Also, the structure of international cooperation in this research shall be taken into consideration. It is interesting to note that the most prolific countries producing these articles are the USA, Italy, China, and the UK (total citations by country). At the same time, the USA, the UK, and South Africa show the highest numbers of citations on the studies considered (total citations by country).

The keywords can well represent the relevant topics assessed in the publications and the keywords-plus considered. It is possible to observe a mixture of different topics related to pandemics and COVID-19 and broadly to Coronavirus. Climate change also got high relevance, signaling as the trendiest keyword. This fact is interesting because it reveals that the literature investigates the outbreak of the pandemic inside the more significant phenomena connected to climate change. At the same time, it is not surprising that one of the most used keywords is China. When it comes to keyword-plus in this line of literature, one shall emphasize the role of humans. More detailedly, central is the role of humans and the human and public health and China. The results are sketched in Appendix A.

Further outputs shall be highlighted from the cluster conceptual maps. The examination of the first cluster renders the following observations:

1. The dynamic social aspects and the medical\epidemiological topics on the right of the axis (emergency service and hospital emergency service are helpful to interpret the axis). At the same time, there appears a hot topic from the blue cluster representing the epidemiological dynamics—the disease outbreak. This encompasses essential elements, such as the organization of healthcare (and leadership) and healthcare quality.
2. On the left, the pandemic's economics emerge—thus, the relevant topics related to the control of the pandemic appear as well.

The different terms are grouped in three distinct clusters. The number is automatically selected on the K-Means procedure (the unsupervised algorithm chosen to group the different terms by evaluating their coordinates). A possible interpretation of the clusters is that the green one is associated with medical topics, the blue one with epidemiological themes, and the red cluster regroups salient socioeconomic issues, which can be reviewed in this literature.

It is worthy to interpret the second axis. Higher values of the axis (at the top of the conceptual structure map) are concepts related to genetics, healthcare systems, food safety, respiratory disease, and meat consumption. These elements are considered undoubtedly hot-button issues for medical reasons and return important messages regarding sustainability and development scholarship. On the other side, it is interesting to note that the lowest values for the axis recall environmental factors like humidity, relative humidity, and environmental impact. These outputs are additional information to corroborate the importance of sustainability and development research within the pandemics and COVID-19 array. These paramount results are saying that different characteristics for the spread of the pandemic exist.

Overall, one can interpret the first axis as the structure of the pandemic, emphasizing the characteristics of the pandemic, and the second axis as the studies focusing on some hot issues on the dynamics.

Of primary order is the left of the first axis, in which one can observe different elements about economic development, environmental policy, sustainability, and urban planning. It is possible to see that these topics are strongly associated with the disease spread. Therefore, air quality, land use, and consumption behavior can acquire cardinality as the disease spreads. Another critical point is artificial intelligence, coupled with classification methods and new technologies.

The robustness study performed on five clusters is beneficial—it checks for a different assumption with respect to the first validated cluster analysis. In particular, the robustness checks are relevant to explore data for a more accurate representation of emerging issues in a smaller number of documents than the initial analysis. For example, there are two emerging topics: artificial intelligence (AI) and gender issues (female) in pandemics. This piece of data also claims for enhanced research on the role of the vulnerable in pandemics in order to better understand them. Appendix A contains a comprehensive summary of the findings of the robustness analysis. The whole outputs are sketched in Appendix B.

## Discussion: Lessons learned—The black swan and the breakthrough

This work scrutinized early research on the existing relationship between COVID-19, Coronavirus, sustainability, and development. The study had the scope of mapping scholarly directions and investigating prospective advances. For this purpose, a merged bibliometric dataset including relevant searches on Coronavirus, COVID-19, sustainability, and development has been investigated. Finally, the paper drew selected short- and long-run policy implications to face the emergency and embrace sustainability and development schemes on these bases.

The bibliometric analyses exploited rendered various significant results: research on COVID-19 concerning past Coronavirus infections sharply increased, underpinned by a considerable bulk of scholarship in sustainability and development subjects. Principal vectors pivoting COVID-19 publications include medical, technical, socioeconomic, and sustainability factors. As a result, this merged database allows exploring the linkages between the pandemic, the environment, health policy, and the valuable organization in disease control.

This research identified the different "core" of the literature, using both exploratory data analysis and multidimensional data analysis techniques. In particular, one can observe three central clusters which seem to classify the trend information in the literature: firstly, epidemiological, medical, and organizational, and health policies found in disease control; secondly, environmental and medical issues; lastly, socioeconomic circumstances which can be prime drivers to the spread of the disease (see Appendix D). These socioeconomic factors ought to be analyzed through a policy lens and allow speculation of relevant research hypotheses. This fact would address the consolidated pulsing development policy request to prioritize the infectious disease fight and public health support within the global development agenda (UN, 2015; WHO, 1996).

The clusters better interpret the literature because they identify the key concepts (see Fig. 1). For instance, in the first cluster on the left, we can consider relevant elements on the transmission dynamics of the virus (disease spread and disease transmission). Following Coccia (2020a), it is possible to observe that the transmission dynamics of viral infections, before-mentioned as COVID-19, are determined by systemic elements, such as general ubiquitous factors (for instance, incubation period and biological features of the virus, etc.). At the same time, special spatial features that are unique to a particular location may be relevant to the transmission dynamics (the author refers to the complex interaction and link within air pollution. Viral infectivity is characterized by specific biological properties characterizing the viral infectivity and also the weather conditions). In addition, individual health levels can be beneficial as the attitudes, immune systems, age, gender, as well as further aspects. In this sense, Coccia (2020a) provides an example of these factors.

According to Coccia (2021), it is possible to observe that wind speeds aid the droplet's dispersion and evacuation. At the same time, in Coccia (2020b) we can observe that the amount of viral agents in the air has decreased, as has the dynamics of viral infection transmission between humans, both of which are beneficial outcomes. In this case, we can observe the “environmental factors” as relevant in the second cluster with a position near the first cluster.

Finally, considering Coccia (2021), extremely short-lived environmental inconsistencies, which can emerge as a result of the interaction of air pollution and weather circumstances, might induce a viral pandemic (epidemic spread), resulting in significant harm to the population's health, the economy, and society as a whole. According to the mentioned studies, levels of air pollution in northern Italian cities, along with limited wind power output, have expedited the COVID-19 spread of the pandemic, which is also leading to increased deaths and cases. These topics are typically observed in both the first and the second cluster.

Ultimately, quarantine can help to significantly reduce the spread of viral infectivity when everything has been said and done. These elements can be observed both on the first and the second cluster. It is interesting to note that these elements in Fig. 1 are near the “viral disease” concept, which means that these elements are typically bounded in the literature (Coccia 2021).

Finally, we can assess “disease control” and “disease outbreak” in the second cluster. Quarantine has been demonstrated to significantly reduce the spread of viral infectivity among humans (Coccia 2020a).

Along with analyzing the concepts in each cluster, we found interesting concepts such as “urban area” and “population density”. The urban area and population density concepts can relate to human mobility. This factor allows the spread of viruses. Therefore, it may be argued that the virus demands a high amount of human interactions to spread over the different territories (Bontempi et al., 2021; Rusciano et al., 2019).

The concept of “sustainability” (near “sustainable development,” which is at the same time close to “environmental factor” and “environmental impact”) should be explained in their position in Fig. 1. Cities in rich and developing nations are plagued by pollution, which is ubiquitous in metropolitan areas (see Coccia, 2020a). A high concentration of air pollution may induce chronic respiratory disorders and increase the susceptibility of individuals to any infectious diseases (Domingo et al., 2020). Furthermore, people who reside in areas with high levels of air pollution are more prone to suffer from respiratory illnesses since particulate matter and infectious organisms such as SARS-CoV-2 are more likely to combine (Coccia, 2020b). It emerges a possible interpretation of the literature suggesting relevant concepts sorting from the statistical analysis. Finally, it is possible to note that the term environmental sustainability is also present in the first cluster. The word is very close to the term health care policy and respiratory disease policy. That means environmental sustainability is the long-run element connecting the environment, health and the economy (Coccia, 2020a Bashir et al., 2021, Praveena and Aris, 2021, Wang and Zhang, 2021. As a result, health care policy should be considered on an economic system that allows for their economic sustainability.

The bibliometric analysis performed synthesizes the existing literature on COVID-19, Coronavirus, development, and sustainability. However, the analysis needs to be addressed to identify the array of policy solutions and alternatives proposed in the reviewed studies. For each issue within the conceptual maps, different trade-offs are identifiable by exploring the existing literature. Relevant examples of trade-offs detected from the conceptual maps are:

1. Social distancing with contact tracing vs. total lockdown (see "disease control").
2. Hospital admission vs. monitoring at the patients' home with a non-severe illness ("organization and management").
3. Vaccine research vs. research on the treatment ("major clinical study").

A relevant point is how to use bibliometrics for decision making. Examples of bibliometric techniques in decision-making are presented in Holden et al. (2005) and Vilchez-Roman et al. (2020). In general, mapping allows the possibility to evaluate the different relationships between variables identified by the studies. Furthermore, the different works allow hypothesizing useful results which can be applied in decision-making models. For instance, in our case, we have identified various trade-offs which can become part of decision-making models.

It shall be noticed that the proximity of the terms on the conceptual map shows that these words appear in the same studies. Thus, the terms and problems are logically interconnected. For this reason, health policies are typically considered to "control the disease" in the context of "disease outbreak". Similarly, health policies require an effort of "organization and management" to be planned.

This gives a chance to foresight different scenarios. As a tentative technique for policy analysis and assessment, each result's significance can be weighted, gauging its number of citations. This way, the ultimate research goal will not solely be the descriptive analysis of the emerging themes in the literature, yielding a proper prescriptive analysis of optimal policies identified within the publications. This paves the way for further investigation possibilities.

The fact that most of the articles have been published in a small number of journals and by a few publishers presents a potential constraint to work. Thus, specific editorial policies of the journal or publisher are likely to have influenced the mixed results—for example, an increase in the number of publications might exaggerate the popularity of various keywords—and that the mixed results are not a coincidence. This inflation might introduce "noise" into the analysis, and as a result, the outcomes should be interpreted with caution in this regard.

The influence of research in the articles under consideration on a given topic is another important factor to be underpinned. Accordingly, research and scientific studies have a number of good social consequences, including enhanced health and prosperity, cultural and economic development, and the advancement of science generally. These experiments have a broader variety of benefits than most others (Greenhalgh et al., 2016). Successful scientific study results may also lead to the development of the most effective processes, which may be employed to accelerate advancement. Innovations may spread and gather pace if they are based on considerable evidence and even more comprehensive research—rather than being constrained by a narrow focus. Product research and development can now be assisted by educational institutions, allowing them to be more effective in their respective fields of study and employment. Science-based work may be used to demonstrate the creation and use of knowledge in this context (Greenhalgh et al., 2016). However, when there are too many factors to account for in the publication (like in the case of Cruz Rivera et al., 2017), it is difficult to quantify the influence of research in the near term.

Bibliometrics provides the opportunity to serve as a guide for healthcare experts and physicians. From the start of the pandemic, a growth in papers on COVID-19 proliferated (Diéguez-Campa et al., 2020). Therefore, bibliometrics can be a relevant source of new knowledge on academic developments, for the study of the diseased, to analyze the pandemic dynamics and health science, and finally, pharmacotherapy (see Thompson and Walker, 2015). It is relevant to assess the extent to which the number of papers helps or hinders the progress of the field; hence, it is necessary to investigate the relationship between the number of works published and the association between socioeconomic, epidemiological, and medical-cum-demographic influences. Following Jung et al. (2021), the exercises in which a result was presented as a working paper or as an extended abstract, even though they had been uploaded on peer-reviewed preprint servers on the websites before study completion, were given less impact and exposure than the others being fully-fledged published. At the same time, there are many reasons for which publications can be marginally prominent during the spread of the pandemic. Not all the published results can adequately reach the majority of the scientists working in that field. Batooli and Sayyah (2020) show that researchers and readers pay close attention to COVID-19 posts. The conclusion of their study considers altmetric and citations identifying a robust positive relationship. Overall, a hypothesis could be that the correlation between the number of publications and the impact of medical, socioeconomic, and epidemiological factors seem slightly apparent in the pandemic period.

From the bibliometrics result, the criticalness of the vulnerable and inequality analyses is also clear. For instance, one of the key themes is gender (female\male in the conceptual map). The concept "female" is strongly related to the theme "health care system", "food safety", and "risk factor". It is interesting that the role of "health care personnel" has a crucial role for both the gender-specific "health care system" and "health care policy "(see Ruggeri et al., 2018). This argument implies a pivotal role for gender policies. It suggests a deep relationship between women in healthcare, which shall emphasize germane sectorial policies and dynamics. For example, one might think about salaries, quality of work, and practices such as balanced life-work ratios—e.g., dedicated part-time work programs. On the other hand, the term "risk factor" corroborates this paper's findings on preparedness. Similarly, the word "food safety" links the importance of food within this multidimensional crisis, revealing intriguing gender dynamics.

## Conclusions: The call for vulnerable-centered policies and health justice

The COVID-19 crisis can be faced by considering short-term and long-term policies related to environmental aspects and, in general, sustainability. It is crucial to plan enhanced coordination at an international level toward environmental sustainability. The general national policies facing up COVID-19 can assist this mandate, sustaining the different economic systems at the same time. However, it is also necessary to consider the effects of the environment in the long run, which can lead to broader unsustainability situations. Health can depend on this and the literature confirmed it. Environmental factors can, indeed, lead to a diffusion of the pandemics and, in general, to adverse conditions for humans. So health policies can also be conceived as a long-run objective on more general environmental policies. In this perspective, health policies may be helpful in improving people's health. More structural approaches can substitute crisis management and social policies in the long term. Developing countries can follow this path on sustainability (see Coccia, 2021a and 2021b; Gouveia and Inglesi-Lotz, 2021; Paramati et al., 2017; Inglesi-Lotz and Ajmi, 2021; Boroujeni et al., 2021).

Health justice can become a tailored resilience tool, of foremost importance for the poor and those living in developing countries. The COVID-19 crisis shows the necessity for higher health justice in these economies. The path of higher sustainability passes through the management of the most relevant interconnections that exists globally. In this optic, the COVID-19 spread is indicative for the multifaceted crises generated by the outbreak and the overall pandemic.

Preliminary evidence also highlights the importance of a solid national health system to tackle the occurring pandemics and adapt to shocks (Legido-Quigley et al., 2020). These eventualities are confirmed by early figures depicting the emergencies or the potential risks faced by some countries—and their citizens—lacking a sound public health system (World Health Organization, 2020b; Abel and Gietel-Basten, 2020). The data becomes dramatic when applied to least-developed countries, featuring significant inequalities and socioeconomic sufferings (Loayza and Pennings, 2020; Phillipson et al., 2020; Sumner et al., 2020). Above all, developing countries will bear the brunt of the crises that originated from the pandemic. COVID-19 is causing even harsher damages to vulnerable households, firms, and communities and is likely to be devastating in the short and long term—as for or even more than precedent pandemics figures (Bapuji et al., 2020; Loayza and Pennings, 2020; Benatar, 2002).

The interconnectedness of AI and gender within the COVID-19, sustainability, and development discourse also arose from the analyses. The former topic underlines the need to encompass AI, the internet of things (IoT), and up-to-date ICT and technologies to monitor, analyze, map, and prevent risks and spread infection (Peeri et al., 2020). The latter theme is of particular importance and confirms the findings of this work, validating the importance of gender studies in disentangling gender vulnerability, the repercussions on women, and the gender gaps and dynamics due to global health and infectious disease (Alon et al., 2020; Wehnham et al., 2020; Flatø et al., 2017; Gilbert and Walker, 2002).

The urgency of the issues been tackled calls for rapid structural socioeconomic reversals. This will need contemplating prompt humanitarian action, risk management, and mitigation, supported by granular and robust data, information, and analyses put forward at national and global levels (Anderson et al., 2020; Peeri et al., 2020; Jonas, 2013). Thus, prompt scrutiny of the socioeconomic consequences of the pandemics is compelling. In contrast, standard development tools for enhancing vulnerable resilience are limited by these interconnected crises—see microfinance tools (Abel and Gietel-Basten, 2020).

More profoundly, the crises demand a resurgence of health justice measures supported by novel public health ethics patterns (Benatar, 2002; Beauchamp and Steinbock, 1999; Beauchamp, 1976). The latter will include access to vaccines, health investments, and public care for all, yet resilience and preparedness primarily targeting both the vulnerable and developing countries (Aldieri et al., 2021; McKibbin and Fernando, 2020; Yamey et al., 2020).

Sustainability is critical for gradually adapting to the new standard, embarking on novel unexplored development pathways and yardsticks. As observed (Di Marco, 2020; Hakovirta and Denuwara, 2020), this might require revisiting the whole sustainable development foundations, yielding centrality to human health and pandemics. Suitable global health futures will be grounded on critical preparedness, readiness, and response actions (WHO, 2020a). Long-sighted, informed guidance for development policy would be in the frontline of this process, rendering desirable scenarios and adaptation to change in light of the pandemic and interconnected crises evolution.

## Appendix A

**Table 1:** Results from the Bibliometric Methodology

**Statistical results**			
*Main Information about data*			
Documents	306		
Sources (Journals, Books, etc.)	168		
Keywords Plus (ID)	1820		
Author's Keywords (DE)	817		
Period	2003 - 2020		
Average citations per documents	9.301		
Authors	841		
Author Appearances	1350		
Authors of single-authored documents	35		
Authors of multi-authored documents	806		
Single-authored documents	52		
Documents per Author	0.364		
Authors per Document	2.75		
Co-Authors per Documents	4.41		
Collaboration Index	3.17		
Document types			
Article	186		
Book	23		
Book Chapter	14		
Data Paper	3		
Editorial	13		
Letter	5		
Note	3		
Review	53		
Short survey	6		
Annual Scientific Production			
Year	Articles		
2003	2		
2005	3		
2006	4		
2007	5		
2008	3		
2009	10		
2010	8		
2011	4		
2012	7		
2013	4		
2014	10		
2015	7		
2016	13		
2017	11		
2018	16		
2019	15		
2020	184		
Annual Percentage Growth Rate	30.47188		
Most Productive Authors			
Articles Authors	Articles Fractionalized		
12 GAUTAM S	6.00		
9 FAYE B	3.00		
9 GALANAKIS CM	3.00		
6 HELM D	3.00		
6 IVANOV D	3.00		
6 SICHE R	3.00		
5 TALLEY NJ	3.00		
4 HAGHSHENAS SS	2.70		
4 PAVESI A	2.12		
4 BCHER M	2.00		
Top manuscripts per citations			
Paper	TC	TCperYear	
1. KRUUK H, 2010, OTTERS: ECOL , BEHAV AND CONSERV	233	21.18	
2. ADGER WN, 2009, FRONTIERS ECOL ENVIR	209	17.42	
3. BALMFORD A, 2005, ECOL LETT	188	11.75	
4. ALVES RRN, 2007, J ETHNOBIOLOGY ETHNOMEDICINE	175	12.50	
5. SMITH MC, 2009, GOAT MEDICINE: SECOND E	168	14.00	
6. JONES JPG, 2008, CONSERV BIOL	136	10.46	
7. MYERS SS, 2009, ANNU REV ENVIRON RESOUR	123	10.25	
8. GREGER M, 2007, CRIT REV MICROBIOL	119	8.50	
9. WOOD JLN, 2012, PHILOS TRANS R SOC B BIOL SCI	98	10.89	
10. THOMAS F, 2007, PARASIT AND ECOSYST	68	4.86	
Corresponding Author's Countries			
Freq	SCP	MCP	MCP_Ratio
22 0.1594	17	5	0.227
15 0.1087	10	5	0.333
11 0.0797	3	8	0.727
10 0.0725	5	5	0.500
7 0.0507	3	4	0.571
6 0.0435	6	0	0.000
6 0.0435	6	0	0.000
6 0.0435	6	0	0.000
6 0.0435	5	1	0.167
5 0.0362	5	0	0.000
SCP: Single Country Publications			
MCP: Multiple Country Publications			
Total Citations per Country			
Country	Total Citations Average	Article Citations	
1. USA	603	27.409	
2. UNITED KINGDOM	593	59.300	
3. SOUTH AFRICA	188	37.600	
4. BRAZIL	175	175.000	
5. FRANCE	114	28.500	
6 CHINA	93	8.455	
7. SWEDEN	51	51.000	
8. CANADA	47	7.833	
9. ITALY	41	2.733	
10. AUSTRALIA	32	4.571	
Most Relevant Sources			
Sources	Articles		
1. INTERNATIONAL JOURNAL OF ENVIRONMENTAL RESEARCH AND PUBLIC HEALTH	17		
2 SUSTAINABILITY (SWITZERLAND)	16		
3. ENVIRONMENT DEVELOPMENT AND SUSTAINABILITY	9		
4. SCIENCE OF THE TOTAL ENVIRONMENT	7		
5. TOURISM REVIEW	6		
6. TRANSPORTATION RESEARCH PART E: LOGISTICS AND TRANSPORTATION REVIEW	6		
7. GLOBAL PUBLIC HEALTH	4		
8. INTERNATIONAL JOURNAL OF ADVANCED SCIENCE AND TECHNOLOGY	4		
9. LANDSCAPE AND URBAN PLANNING	4		
10. BULLETIN OF ENVIRONMENTAL CONTAMINATION AND TOXICOLOGY	3		
Most Relevant Keywords			
Author Keywords (DE)	Articles	Keywords-Plus (ID)	Articles
1. COVID 19	69	HUMAN	74
2. CORONAVIRUS	37	ARTICLE	61
3. PANDEMIC	18	CHINA	45
4. SARS COV 2	16	HUMANS	45
5. CLIMATE CHANGE	15	PUBLIC HEALTH	40
6. CHINA	13	CORONAVIRUS	37
7. SUSTAINABILITY	13	ANIMALS	33
8. FOOD SAFETY	8	SUSTAINABLE DEVELOPMENT	33
9. DISASTER MANAGEMENT	7	CORONAVIRUS INFECTION	32
10. FOOD SECURITY	7	NONHUMAN	32

## Appendix B – Clustering of the topics

The last step of this research was to examine the multidimensional data analysis from the publication bipartite network obtained from the bibliometric database. In order to carry out the analysis, the data matrix scrutinized the different keywords specified by the authors for each study. Then we have performed a multiple correspondence analysis to represent the conceptual structure of the investigating literature (Aria and Cuccurullo, 2017; Greenacre and Blasius 2006). The procedure started by extracting the first two axes, which are maximizing the available information. On the first axis, we were able to identify and interpret the main structural topics linked with the socioeconomic development of the pandemic. This procedure returned a first clustering validation of the topics—that entailed three clusters—and the second block of clusters that returned five subjects. The latter has been used as a sensitivity analysis of the techniques operated to prove the study's validity.

## Appendix C – Multiple Correspondence Analysis (MCA

Multiple Correspondence Analysis is an approach to extending correspondence analysis to examining two or many categorical variables (Greenacre and Blasius 2006, Gilula et al. 2001 Guttman 1941). Here we show a description of the multiple correspondence analysis (MCA). Following Abdi and Valentin (2007), MCA is a statistical technique for analyzing a collection of measurements represented by a set of nominal variables. Different levels describe these variables, which can be directly coded in binary variables. Following Husson (2021), the analysis aims to study both the individuals and the variables. In particular, we want to study the inter-individual heterogeneity and the similarity between the different statistical units considered. At the same time, we want to study the relationships between the different variables, particularly considering their categories. It is possible to visualize the different associations 819 between the differentcategories of the variables. Finally, a relevant aim of the MCA is the construction of a set of variables obtained as a synthesis from the initial qualitative variables. According to the findings of Abdi and Valentin (2007), we may interpret our data in the following way. Since the values of different nominal variables are near together, they seem to emerge in the same observation simultaneously. Likewise, since the thresholds are close together, the clusters of findings correlated with such two levels are also identical. The representations of the different clusters or groups can be observed in the different convex hulls (Aria and Cuccurullo 2017). The convex hulls allow representing the concepts that are considered explicitly in the same papers.

It is possible to identify a reduced distance on the map between two or more concepts, which indicates that papers in the literature are more likely to reference the articles together in cases where the distance is shorter (Gherghi and Lauro 2004). In this sense, the different positions of the concepts reflect the coordinates of the elements. Furthermore, some concepts are nearer to others, which means the papers consider the nearest concepts jointly.

## Appendix D

**Table 2:** Table with the cluster interpretation

Cluster 1	Epidemiological, medical, and organizational and health policies in disease control	red
Cluster 2	Environmental and medical issue	green
Cluster 3	Socioeconomic circumstances	blue
